# Combination of Ashwagandha Water Extract and Intermittent Fasting as a Therapy to Overcome Cisplatin Resistance in Breast Cancer: An *in vitro* and *in vivo* Study

**DOI:** 10.3389/fnut.2022.863619

**Published:** 2022-07-04

**Authors:** Sajidah Jawarneh, Wamidh H. Talib

**Affiliations:** Department of Clinical Pharmacy and Therapeutics, Applied Science Private University, Amman, Jordan

**Keywords:** breast cancer, nutritional intervention, Ashwagandha, intermittent fasting, multidrug resistance, cisplatin, apoptosis, Warburg effect

## Abstract

Breast cancer is considered a universal public health dilemma in women. Due to the high toxicity and low selectivity of conventional anticancer therapies, there is a growing trend of using plant-derived natural products in cancer prevention and therapy. Ashwagandha (*Withania somnifera*, WS) has been used in the Mediterranean region and Ayurvedic medicine for millennia as a functional food and a medicinal plant with anticancer activity. Besides, intermittent fasting (IF) has been engaged recently in cancer treatment. Hence, the combination of WS and IF provides possible solutions to treat cancer and reduce chemoresistance when combined with chemotherapy. In this study, WS root (WSR), IF, and cisplatin were tested on cisplatin-sensitive (EMT6/P) and cisplatin-resistant (EMT6/CPR) mouse mammary cell lines. The phytochemical content of the WSR extract was analyzed using liquid chromatography–mass spectrometry (LC-MS) analysis. Antiproliferative and apoptotic effects were assessed for WSR extract, cisplatin, and their combination *in vitro* using [3-(4,5-dimethylthiazol-2-yl)-2,5-diphenyl-2H-tetrazolium bromide] (MTT) and caspase-3 assays. An *in vivo* study was used to assess the effect of WSR extract, IF, cisplatin, and their combinations in mice inculcated with EMT6/P and EMT6/CPR cells. The safety profile was also investigated using liver enzymes and creatinine assays. *In vitro*, WSR extract and cisplatin had a synergistic effect in both cell lines. The same combination induced an apoptotic effect higher than the single treatment in both cell lines. *In vivo*, several combinations of WSR extract, IF, or cisplatin caused significant tumor size reduction and improved the cure rate in mice implanted with EMT6/P and EMT6/CPR cell lines. IF-treated groups showed a significant reduction in serum glucose and an elevation in β-hydroxybutyrate (BHB) levels. In the safety profile, WSR extract, IF, and their combinations were safe. Overall, the combination of WSR extract and IF provides a promising solution for breast cancer treatment besides cisplatin by reducing the proliferation of cancer cells through induction of apoptosis. Moreover, they minimize cisplatin toxicity to the liver and kidney.

## Graphical Abstract

Summary of the effect of Ashwagandha and intermittent fasting on cisplatin resistance breast cancer *in vitro* and *in vivo*. Created by Sajidah Jawarneh. 
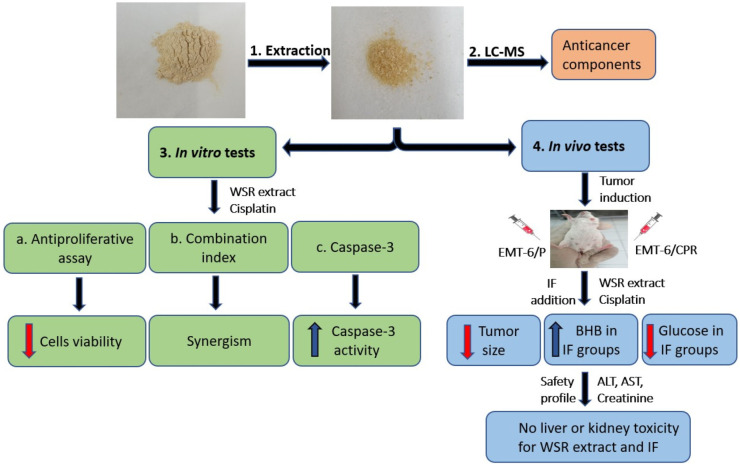

## Introduction

Breast cancer is a major public health problem because it is the second cause of death and the most common cancer in women worldwide. Resistance to cancer treatments plays a major role in breast cancer issue exacerbation (1). As an example, cisplatin is uniformly used in cancer treatment; nevertheless, its use is limited because of serious side effects and resistance (2). Resistance is divided into primary drug resistance and multidrug resistance (MDR) (3). To clarify, MDR is the resistance of cancerous cells to various anticancer drugs with different structures and mechanisms of action. Chemoresistance has several molecular mechanisms, such as deregulation of apoptosis, deregulated autophagy, enhanced DNA damage repair, and p53 inactivation (4).

Apoptosis is the natural mechanism for programmed cell death. Apoptosis has two major pathways: extrinsic and intrinsic. In the extrinsic pathway, the death legends (e.g., TNF and Fas-L) activate the formation of a death-inducing signaling complex (DISC). This results in the formation of caspase-8 and –10 followed by executioner caspases-3, –6, and –7 activation (5). In the intrinsic pathway, different apoptotic stimuli upregulate BCL-2 homology domain 3 (BH3)-only proteins, which activate BCL-2 homology domain 3 (BH3) and BCL-2 homology domain 3 (BH3), releasing cytochrome-c. Then, cytochrome-c facilitates the conversion of procaspase-9 to caspase-9, which can activate the executioner caspases-3 and –7. The latter start to hold up proteins leading to cell death (6). Therefore, overexpression of oncogenes mediates the inhibition of apoptosis and that leads to the suppression of p53, enhancement of antiapoptotic proteins, such as B cell CLL/lymphoma-2 (Bcl-2), and downregulation of pro-apoptotic proteins like caspases, Bcl-XL/Bcl-2-associated death promoter (Bad), and BAX/BAK. Therefore, several researchers have targeted caspases to overcome resistance to chemotherapy (5).

Nutrition interventions have valuable effects in terms of cancer prevention and treatment (7). For example, Ashwagandha has been used as an indispensable plant in the Mediterranean region and in Ayurvedic medicine for millennia (8) as a functional food due to its immense nutritional value with various biological effects like cancer (9). Comparatively, intermittent fasting (IF) has been engaged in the newly developing treatment approaches because of its benefit in fighting cancer (10).

Ashwagandha (*Withania somnifera*, WS) belongs to the family Solanaceae and has been used as an antitumor, anti-inflammatory, antidiabetic, antistress, hepatoprotective, and nephroprotective agent (11). WS root (WSR) has many active constituents, such as alkaloids, flavonoids, withanolides (e.g., withaferin A and withanone), and succinic acid (11, 12). WSR extract can promote apoptosis in breast cancer through caspase-3 activation and downregulation of the antiapoptotic protein Bcl-2 (13). Additionally, it enhances the efficacy of both chemotherapy and radiotherapy (14).

There is growing attention to harnessing IF to minimize tumor growth and improve cancer treatment efficacy. IF mimics Ramadan fasting in Islamic countries, which is applied in many of the Mediterranean region’s countries (15). It reduces glucose levels and affects glucose metabolism (glycolysis) inside the cell by reversing the Warburg effect (16). IF depends on calorie restriction or fasting over an extended period (e.g., 16–48 h). This time is enough to activate ketogenesis increasing ketones [e.g., β-hydroxybutyrate (BHB) and acetoacetate], mitochondrial stress resistance, antioxidant defense, and autophagy (17). Currently, IF is used as an adjunct therapy for cancer along with chemotherapy. When used for short terms, it enhanced the chemotherapeutic effect. Moreover, short terms use of fasting showed elevation in oxidative stress and DNA damage causing induction of apoptosis (10).

In this perspective, the Warburg effect (aerobic glycolysis) represents an essential hallmark of cancer, since cancer cells have accelerated glycolysis and exaggerated lactate production, even under fully oxygenated conditions. This ends with oxidative stress reduction and consequently, resistance to diverse factors including apoptosis and cytotoxic drugs. In the bargain, lactate production produces an acidic environment, which is compatible with proliferation and metastasis (18).

A combination of herbal extract with fasting and a triple combination of herbal extract, fasting, and chemotherapy has not been evaluated in the literature to treat cancer. Accordingly, this study was designed to test the effect of WSR extract with IF with cisplatin as a new combination therapy to overcome cisplatin-resistant breast cancer in a mouse model.

## Materials and Methods

### Extract Preparation

**FIGURE 1:**
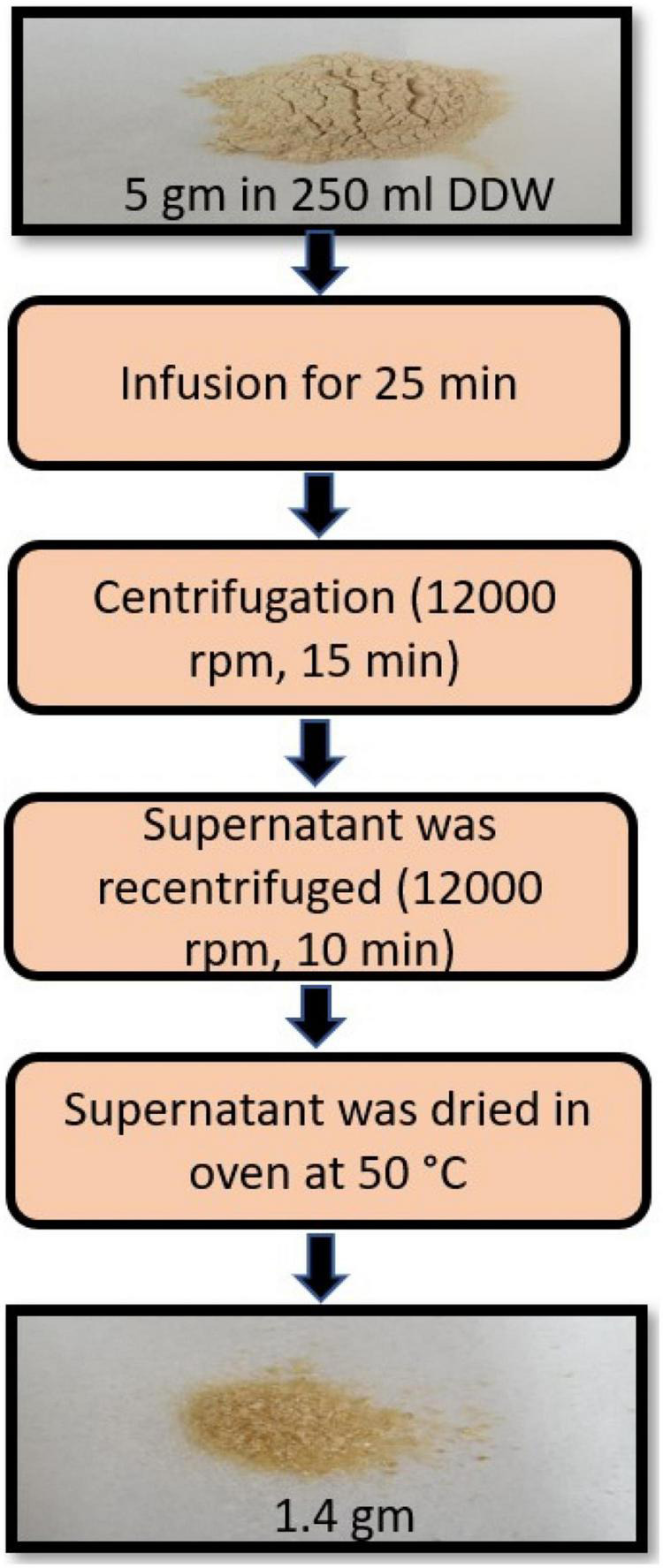
Scheme for extraction of water-soluble material from the *Withania somnifera* root (WSR).

Powdered WSR was purchased from Zokiva Nutritionals, US. The extract preparation was performed following the protocol previously optimized by Kumar et al. (19). Briefly, 5 gm of the powdered root was infused in 250 ml (1:50 w/v) of freshly boiled double distilled water (DDW) for 25 min. After that, the infusion was left to cool to room temperature and centrifuged (12,000 rpm or 8050 X g, 15 min). The supernatants were recentrifuged (12,000 rpm, 10 min) (19). The supernatants were then dried in the oven at 50°C (20). The yield of the dried aqueous extract was 1.4 g (Figure 1).

### Liquid Chromatography–Mass Spectrometry Analysis

The preparation of the sample was carried out by dissolving 20 mg of WSR extract in 2 ml DDW and then completed with acetonitrile to 50 ml. The sample was centrifuged at 4,000 rpm for 2 min. The autosampler was then loaded with 1 ml of the sample and the injection volume was 3 μl. The instrument was utilized by Ion Source Apollo II ion funnel electrospray source with the following characteristics [dry gas flow 8 l/min; capillary voltage: 2500 v; nebulizer gas: 2 bar; dry temperature: 200°C; mass accuracy: < 1 ppm; mass resolution: 50,000 FRS (Full Sensitivity Resolution); the time-of-flight (TOF) repetition rate: up to 20 kHz]. The separation was accomplished *via* a Burker solo 2-C-18 ultra-high performance liquid chromatography (UHPLC) column (100 mm × 2.1 mm × 2 μm) at a flow rate of 0.51 ml/min and a column temperature of 40°C. All standards were used for the identification of m/z and the retention time. The analysis was performed by Burker Daltonik (Bremen, Germany) impact II ESI-Q-TOF system provided with Burker Dalotonik Elute UPLC system (Bremen, Germany) used for screening compounds of interest.

### *In vitro* Experiments

#### Cell Lines and Culture Conditions

Two mouse mammary cell lines were used in this study: the parent (EMT6/P) and cisplatin resistance (EMT6/CPR) cell lines were purchased from the European Collection of Authenticated Cell Cultures (ECACC; Salisbury, United Kingdom). Cells were grown in minimal essential medium (MEM) supplemented with 10% fetal bovine serum, 1% L-glutamine, 0.1% gentamicin, 1% penicillin-streptomycin solution, and 0.1% non-essential amino acids. Perfect cell culture conditions were provided for cell growth using complete tissue culture media (MEM). All cell lines were incubated in a carbon dioxide (CO2) incubator at 37°C, with 5% CO2, and 95% humidity.

#### Antiproliferative Assay (MTT)

The antiproliferative activity was detected using MTT (the tetrazolium salt, 3,[4,5-dimethylthiazol-2-yl]-2,5-diphenyl-tetrazolium bromide; Sigma, Saint Lucia, United States). The mouse mammary cell lines (EMT6/P and EMT6/CPR) were cultured overnight. Cells were collected using the trypsinization technique, and the exponentially growing cells were counted using the trypan blue exclusion method. After that, the cells were seeded at 10,000 cells/well in 96-well tissue culture flat-bottom microplates for 24 h incubation. After seeding, both the cell lines were exposed to different concentrations of WSR extract (0.39–50 mg/ml) for 48 h. They were also exposed to cisplatin (0.8–100 μM) for 48 h.

In combination treatment, EMT6/P cells were exposed to increasing concentrations of WSR extract (0.01–1.5 mg/ml) with a fixed dose of cisplatin (10 μM) in EMT6/P. In the resistant cell line, cells were exposed to the extract with different concentrations (0.02–2 mg/ml), with a fixed concentration of cisplatin (27 μM). The reduced MTT was assayed at 550 nm using a microplate reader (Biotek, Winooski, VT, United States). Percentage cell survival was calculated for all treatments and compared with untreated cells.

The combination index (CI) was calculated for the WSR extract and cisplatin combination using the previous equation (21), and the resistance fold (RF) was calculated using the following formula (22):


$$C\,I={\left(D\right)}_{1}/{\left(D\,x\right)}_{1}+{\left(D\right)}_{2}/{\left(D\,x\right)}_{2}+\alpha \,{\left(D\right)}_{1}\,{\left(D\right)}_{2}/{\left(D\,x\right)}_{1}\,{\left(D\,x\right)}_{2}$$

Where: (Dx)_1_ = The half-maximal inhibitory concentration (IC_50_) of WSR extract alone (D)_1_ = IC_50_ of WSR extract in combination with cisplatin (Dx)_2_ = IC_50_ of cisplatin alone(D)_2_ = IC_50_ of cisplatin in combination with WSR extract

α = 0 for mutually exclusive or 1 for mutually nonexclusive interaction. Depending on the literature review, both WSR extract and cisplatin exert their anticancer effect by different mechanisms of action. Hence, we applied the mutually nonexclusive model, where α = 1.

CI values are explained according to the following:

CI > 1.3 = antagonism,

CI = 1.1–1.3 = moderate antagonism,

CI = 0.9–1.1 = additive effect,

CI = 0.8–0.9 = slight synergism,

CI = 0.6–0.8 = moderate synergism,

CI = 0.4–0.6 = synergism,

CI = 0.2–0.4 = strong synergism


$$R\,F=I\,C_{50}\,o\,f\,r\,e\,s\,i\,s\,t\,a\,n\,t\,c\,e\,l\,l\,s/I\,C_{50}\,o\,f\,p\,a\,r\,e\,n\,t\,a\,l\,c\,e\,l\,l\,s$$

#### Measuring Apoptosis Induction in Cultured Cells

The caspase-3 assay was used to determine the apoptotic effect of WSR extract and cisplatin in parent and drug resistance cell lines. EMT6/P flasks were treated with IC_50_ concentrations of WSR extract (2.9 mg/ml), cisplatin (positive control; 20 μM), and combination treatment of the extract and cisplatin (0.54 mg/ml + 10 μM, respectively). EMT6/CPR flasks were treated with IC_50_ concentrations of the extract (3.8 mg/ml), cisplatin (positive control; 54 μM), and combination treatment of the extract and cisplatin (0.66 mg/ml + 27 μM, respectively). MEM was used as the negative control. The flasks were then incubated for 48 h. After treatment, cells were collected, washed, and lysed using lysis buffer. Caspase-3 activity was measured using the procedure provided in the standard kit (Caspase-3 Assay Kit, My BioSource, United States).

### *In vivo* Experiments

#### Mice

Forty-two female Balb/C mice weighing 21–25 g (4–6 weeks old) were used in this study. Mice were supplied by the animal house in the Applied Science Private University, Amman, Jordan. All protocols of animal experiments were validated by the Research and Ethical Committee of Applied Science University with Standard ethical guidelines. The animals were kept in separate cages with bedding of wooden shavings. The provided conditions in the animal house included stable temperature at 25°C, 50–60% humidity, continuous air ventilation, and alternating light/dark cycles of 12 h.

#### Tumor Inoculation

Exponentially growing EMT6/P and EMT6/CPR cells were harvested by trypsinization and were washed and resuspended in MEM, at a density of 1.5 × 10^6^ cells/ml. After that, viability was detected using the trypan blue exclusion method. A tumor induction dose of 1.5 × 10^5^ cells in 0.1 ml medium was injected into the abdominal area of each female BALB/C mouse subcutaneously and maintained for 14 days to grow and form new tumors.

#### Mice Groups, Treatment, and Antitumor Activity

**FIGURE 2:**
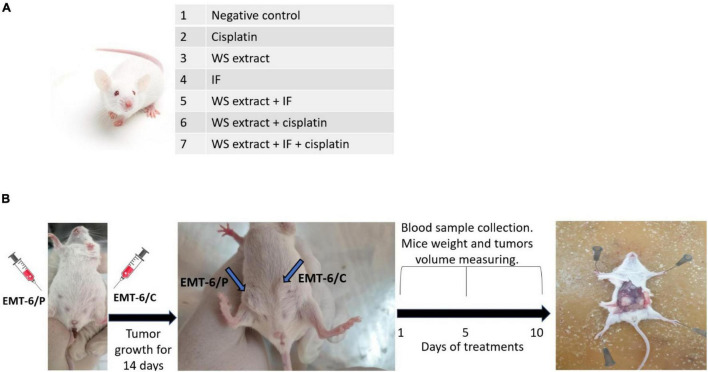
*In vivo* mice study. **(A)** Groups of cisplatin-sensitive (EMT6/P) and cisplatin-resistant (EMT6/CPR) inoculated mice. Treatments were: WSR extract (100 mg/kg/d) by gavage, a daily 18 h of intermittent fasting (IF) and 6 h of eating or/and cisplatin (5 mg/kg/week) by intraperitoneal injections for 10 days. **(B)** Scheme for mice experiment (*n* = 6).

Each mouse was inoculated with EMT6/P on the right side and EMT6/CPR on the left side. The treatments were started 14 days after tumor inoculation. Forty-two tumor-bearing mice were used in this investigation, and the mice were divided into seven groups (*N* = 6 for each group; Figure 2A). Group 1 was used as a negative control and was exposed to intraperitoneal injection of the vehicle (phosphate-buffered saline, PBS) of 0.1 ml daily. Group 2 was treated with cisplatin (5 mg/kg/week) by intraperitoneal injections (23). Group 3 was treated with a daily dose of WSR extract (100 mg/kg/d; 24) by gavage. Group 4 was exposed to a daily 18 h of IF and 6 h of eating (*ad libitum* nutrition). Group 5 was treated with WSR extract and IF combination. Group 6 was treated with WSR extract and cisplatin combination. Group 7 was treated with the triple combination therapy of WSR extract, IF, and cisplatin. The treatment lasted for 10 days. During treatment, blood samples, mouse weight, and tumor volumes were taken at three time-points over the treatments on days 1, 5, and 10 (Figure 2B).

Tumor dimensions were measured using a digital caliper. The following formula was used to calculate the tumor volumes (22):


$$Tumorvolume=L\times W^{2}\times 0.5$$

where L = length of the longest aspect of the tumor,

W = length of the tumor aspect perpendicular to L.

Finally, mice were killed by cervical dislocation. The tumors were removed, weighed, and stored in 10% formalin to preserve their morphology.

#### Evaluation of Serum β-Hydroxybutyrate and Serum Glucose Levels

Blood levels of glucose and BHB were assessed on days: 1, 5, and 10, and compared with normal-untreated mice bearing no tumor. Blood glucose levels were measured using the Accu-Chek blood glucose monitoring system (Roche, Basel, Switzerland). BHB Assay Kit (Sigma, United States) was used to measure the levels of BHB in the serum.

#### Evaluation of Liver and Kidney Function in Treated Mice

The level of toxicity exerted by different treatments on the liver and kidney was assessed. Serum levels of alanine aminotransferase (ALT), aspartate aminotransferase (AST), and creatinine were evaluated for WSR extract, IF, cisplatin, and their combinations in addition to the negative control and normal-untreated group. After a serum sample collection, ALT and AST were tested using ALT/GPT kit, AST/GOT kit, and a creatinine assay kit purchased from (BioSystems, Barcelona, Spain).

### Statistical Analysis

Statistical analyses were performed using SPSS (Statistical Package for the Social Science, Chicago, IL, United States 25). All values were expressed as mean ± SEM. IC_50_ values were statistically analyzed using nonlinear regression. Statistical significance among the mice groups was determined using a one-way analysis of variance (ANOVA; *post hoc* test: Tukey). A probability level less than 0.05 (*p* < 0.05) represented a significant difference among groups. *In vivo*, six mice per group were used and statistics were conducted using *n* = 3 or *n* = 6 in the different tests.

## Results

### Liquid Chromatography-Mass Spectrometry Analysis of WS Root Water Extract

**FIGURE 3:**
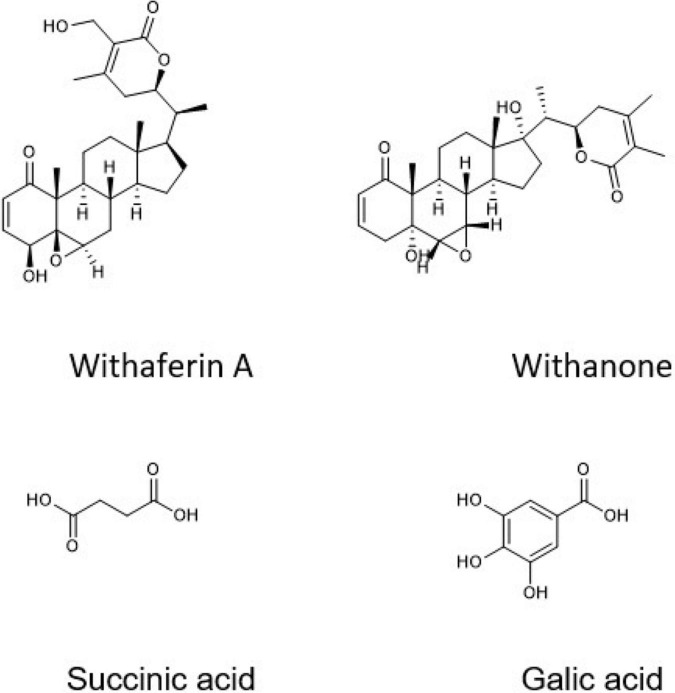
Chemical structure for selected compounds in the water extract of WSR.

According to Liquid chromatography-mass spectrometry (LC-MS) analysis, WSR water extract contained succinic acid (68.52%) as a major compound, and other compounds were found in different concentrations, such as anthranilic acid (16.87%), gallic acid (7.52%), chlorogenic acid (2.81%), and 3,5-dimethoxy-4-hydroxy acetophenone (1.29%; Figure 3). The rest of the compounds were less than 1%, such as vanillic acid, protocatechuic aldehyde, caffeic acid, ferulic acid (trans), apiin, and salicylic acid, rutin, spiraeoside, kaempferol, and isorhamnetin (Table 1).

**TABLE 1:** Liquid chromatography-mass spectrometer (LC-MS) analysis of *Withania somnifera* root (WSR) water extract.

No.	Compound	Molecular formula	RT (min)	Amount (%)
1	Succinic acid	C4H6O4	0.98	68.52
2	Gallic acid	C7H6O5	1.04	7.52
3	Protocatechuic aldehyde	C7H6O3	2.09	0.01
4	Chlorogenic acid	C16H18O9	2.88	2.81
5	Vanillic acid	C8H8O4	3.2	0.18
6	Caffeic Acid	C9H8O4	3.27	0.28
7	Anthranilic acid	C7H7NO2	4.07	16.87
8	Apiin	C26H28O14	5.11	0.01
9	Ferulic acid (trans)	C10H10O4	5.13	0.40
10	Rutin	C27H30O16	5.58	0.40
11	3,5-Dimethoxy-4-hydroxy acetophenone	C10H12O4	5.63	1.29
12	Salicylic acid	C7H6O3	5.78	0.01
13	Spiraeoside	C21H20O12	5.78	0.81
14	Kaempferol	C15H10O6	10.13	0.07
15	Isorhamnetin	C16H12O7	10.51	0.32

### *In vitro* Results

#### Antiproliferative Effect of WS Root Extract, Cisplatin, and Their Combination

**FIGURE 4:**
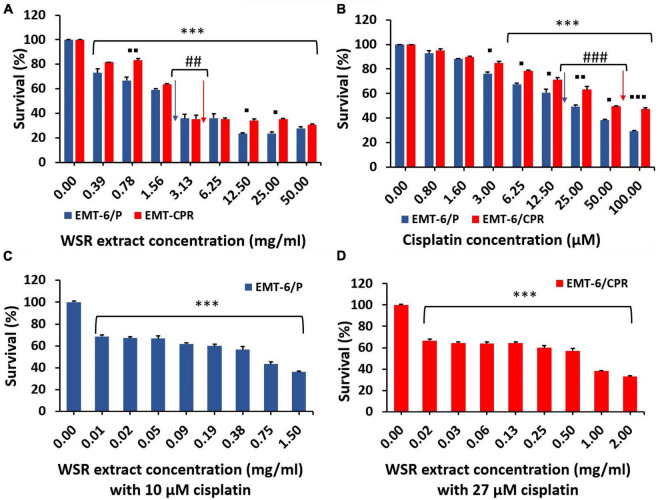
Antiproliferative effect of WSR extract, cisplatin, and their combination against EMT6/P and EMT6/CPR cell lines. **(A)** Treatment of both cell lines with an increasing concentration of WSR extract. **(B)** Treatment of both cell lines with an increasing concentration of cisplatin. **(C)** Antiproliferative activity of various concentrations of WSR extract with 10 μM cisplatin against EMT6/P cell lines. **(D)** Antiproliferative activity of various concentrations of WSR extract with 27 μM cisplatin against EMT6/CPR cell lines. Data are presented as mean ± SEM (*n* = 3). *P < 0.05, ^**^P = 0.001, ^***^P < 0.001. ^##^*P* = 0.001, ^###^*P* < 0.001. ^◼^*P* < 0.05, ^◼◼^*P* = 0.001, ^◼◼◼^*P* <0.001. (*: treatments groups compared with the control (0.00 μM), ◼: EMT6/P survival compared with EMT6/CPR survival, ^#^: survival of EMT6/P at IC_50_ compared with the survival of EMT6/CPR at IC_50_ concentration).

MTT assay was conducted to evaluate the antiproliferative activity of WSR extract, cisplatin, and their combination on cisplatin-sensitive (EMT6/P) and cisplatin-resistant (EMT6/CPR) cell lines. Single treatment of WSR extract or cisplatin attenuated cell proliferation compared with the vehicle control in a concentration dependant pattern as observed in Figures 4A,B. Our results revealed that EMT6/CPR cells presented higher survival rates compared to EMT6/P cells when exposed to the same concentrations of WSR extract or cisplatin. Additionally, EMT6/P and EMT6/CPR cell lines were treated with different concentrations of WSR extract and a fixed concentration of cisplatin. The results showed that this combination significantly concentration-dependently inhibited cell viability (Figures 4C,D).

The analysis of the CI showed that the combination treatment had a moderate synergistic effect employed on EMT6/P and EMT6/CPR cells (CI = 0.78 and 0.76, respectively; Table 2). The mean IC_50_ values are reported in Table 2, and the IC_50_ values of the extract were 2.9 ± 0.18 and 3.8 ± 0.09 mg/ml in EMT6/P and EMT6/CPR, respectively, with an RF of 1.31, which means that EMT6/CPR cells were 1.31 times more resistant to the extract than EMT6/P. Oppositely, IC_50_ of cisplatin was found to be 20 ± 0.5 μM in the EMT6/P cell line and 54 ± 0.08 μM in the EMT6/CPR cell line, which means that EMT6/CPR cells were 2.7 times more resistant to cisplatin in contrast to EMT6/P cells. Thus, a higher concentration of WSR extract or cisplatin is needed to kill 50% of the EMT6/CPR cell line. On the other hand, in the combination, IC_50_ was 0.54 ± 0.011 mg/ml WSR extract and 10 μM cisplatin in EMT6/P cells and 0.66 ± 0.05 mg/ml WSR extract with 27 μM cisplatin in EMT6/CPR cells. As observed, EMT6/P cells were more susceptible to the combination at lower doses than EMT6/CPR being a resistant cell line that has mechanisms to resist the applied combination. The RF of the combination (1.22) is lower than the RF of either WSR extract or cisplatin single treatment (1.31 and 2.7, respectively), which indicates that the WSR extract sensitized the resistant cells to cisplatin.

**TABLE 2:** IC_50_ values for the extract and cisplatin in cisplatin-sensitive (EMT6/P) and cisplatin-resistant (EMT6/CPR) cell lines along with the combination index, related interpretation, and resistance fold.

Cell line	IC_50_ of WSR extract (mg/mL)	IC_50_ of cisplatin (μM)	WSR extract IC_50_ in combination (mg/mL)	Cisplatin IC_50_ in combination (μM)	CI	Interpretation
EMT6/P	2.9 ± 0.18	20 ± 0.5	0.54 ± 0.011	10	0.78	Moderate synergism
EMT6/CPR	3.8 ± 0.09	54 ± 0.08	0.66 ± 0.05	27	0.76	Moderate synergism
RF	1.31	2.7	1.22	2.7		

*Data are presented as mean ± SEM (n = 3). CI, combination index; RF, resistance fold.*

#### Apoptotic Activity of WS Root Extract, Cisplatin, and Their Combination

**FIGURE 5:**
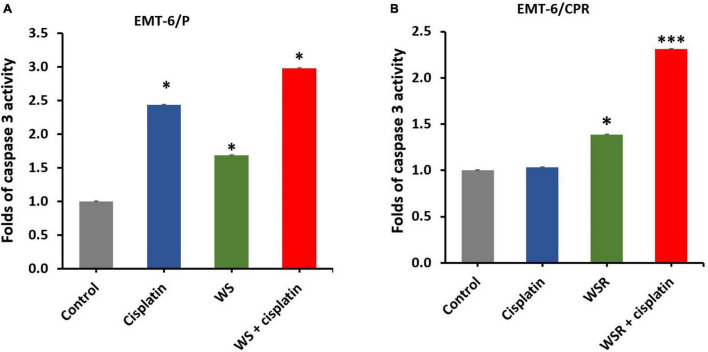
Caspase-3 activity in breast cancer cells after treatment with WSR extract and cisplatin. **(A)** Treatment of EMT6/P cells with cisplatin (20 μM), WSR extract (2.9 mg/ml), and their combination (10 μM cisplatin with 0.54 mg/ml WSR extract). **(B)** Treatment of EMT6/C cells with cisplatin (54 μM), WSR extract (3.8 mg/ml), and their combination (27 μM cisplatin with 0.66 mg/ml WSR extract) in EMT6/C cell line. Results are expressed as means ± SEM (*n* = 3). *P < 0.05, ^***^P < 0.001. (*: treatments groups compared with the control group).

Caspase-3 activity was performed using a caspase-3 assay kit to evaluate the apoptotic effect of WSR extract, cisplatin, and their combination in EMT6/P and EMT6/CPR cell lines. Results of the analysis in EMT6/P cells indicated a significant difference between WSR extract, cisplatin, and their combination compared to control (*p* < 0.05) and between the treatment groups themselves (*p* < 0.05). The combination exhibited 2.98 folds increase in caspase-3 activity compared to the control (Figure 5A). In contrast, the detected results in EMT6/CPR revealed the existence of a significant difference between WSR extract and its combination with cisplatin compared with the control group. As shown in Figure 5B, WSR extract single treatment achieved 1.39 folds increase in caspase-3 activity compared to the control (*p* < 0.05). Furthermore, the combination of WSR extract and cisplatin achieved 2.31 folds increase in caspase-3 activity compared to the control group with a significant difference (*p* < 0.001) compared to the cisplatin-treated group, which showed an insignificant response to cisplatin (*p* = 0.286).

### *In vivo* Results

#### Antitumor Effect of WS Root Extract, Intermittent Fasting, Cisplatin, and Their Combinations

Based on the results of the *in vitro* assay, WSR extract was selected in addition to IF, cisplatin, and their combinations for further evaluation to assess the antitumor activity in Balb/C female mice. According to the results in Table 3, the treated groups showed a significant reduction (*p* < 0.05) in tumor size compared with the negative control, which registered an increase in tumor size of 88.87%. Noteworthy, the triple combination of cisplatin, WSR extract, and IF recorded the highest percentage in the size reduction (100%) and curable rate (100%). Besides, the combination of WSR extract and IF recorded the lowest percentage of size reduction (60.52%) among the combination treatments, along with a curable rate of 50%. The combination of WSR extract and cisplatin registered a reduction in tumor size of 81.12% with a curable rate of 50%. As observed, the same treatments were applied to EMT6/CPR cells. Tumor size was reduced remarkably (*p* < 0.05) for all treated groups as opposed to the negative control, which showed an increase in tumor size of 60.02% from the initial tumor size. Interestingly, combination treatments had higher tumor size reduction than single treatments. Regarding triple treatment, it registered a complete reduction in tumor size (100%); therefore, there were no mice with detectable tumors (100%). On the flip side, the WSR extract and cisplatin combination exhibited a higher reduction in tumor size than the WSR extract and IF combination (69.49 and 53.36%, consequently), besides the same curable rate (66.66%).

**TABLE 3:** Results of WSR extract, intermittent fasting (IF), cisplatin and their combinations concerning tumor size changes, percentage of changes in tumor size, and average tumor weight in EMT6/P and EMT6/CPR cell line.

Treatment group	Av. initial tumor size (mm^3^)	Av. final tumor size (mm^3^)	(%) change in tumor size	(%) mice with no detectable tumor	Av. tumor weight (gm)
***EMT6/P***					
Control	358.05 ± 35.02	676.24 ± 170.41	88.87	0	0.54 ± 0.172
Cisplatin	291.80 ± 12.86	75.25 ± 4.77	–74.21	50	0.066 ± 0.004
WSR	367.13 ± 46.79	176.34 ± 33	–51.97	33.33	0.151 ± 0.021
IF	381.63 ± 24.84	180.33 ± 19.30	–52.75	33.33	0.127 ± 0.023
WSR + IF	330.25 ± 18.14	130.37 ± 12.87	–60.52	50	0.105 ± 0.007
WSR + cisplatin	389.23 ± 21.22	75.50 ± 7.72	–81.12	50	0.069 ± 0.001
WSR + IF + cisplatin	351.42 ± 37.10	0.0	–100	100	0.0
***EMT6/CPR***					
Control	215.38 ± 15.57	344.65 ± 64.71	60.02	33.33	0.275 ± 0.04
Cisplatin	200.54 ± 34.27	162.84 ± 17.19	–18.80	33.33	0.132 ± 0.026
WSR	248.48 ± 26.55	147.88 ± 4.96	–40.49	50	0.135 ± 0.008
IF	210.35 ± 23.37	132.06 ± 9.30	–37.22	33.33	0.085 ± 0.009
WSR + IF	246.99 ± 16.34	115.20 ± 3.30	–53.36	66.66	0.08 ± 0.001
WRS + cisplatin	247.23 ± 21.63	75.42 ± 3.21	–69.49	66.66	0.05 ± 0.001
WSR + cisplatin + IF	212.85 ± 22.57	0.0	–100	100	0.0

*Av., average; mmł, cubic millimeter; gm, gram. Data are expressed as means ± SEM (N = 6).*

**FIGURE 6:**
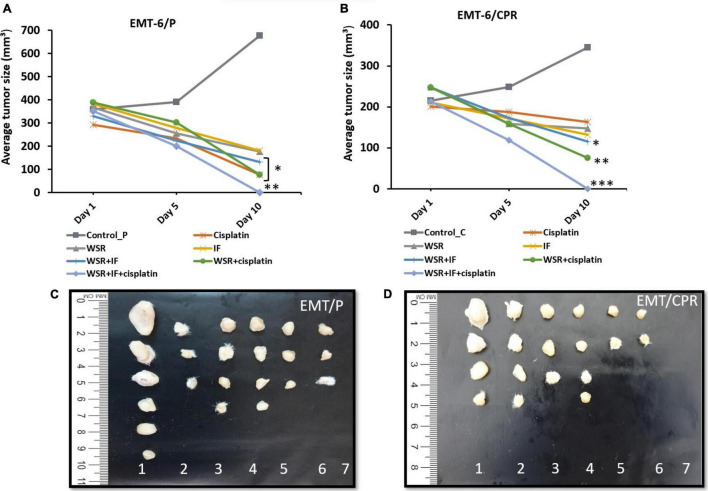
Effect of treatments on tumor size in vivo. **(A)** A plot of change in average tumor size (mmł) vs. time (days) of treatment in the EMT6/P cell line. **(B)** A plot of change in average tumor size (mmł) vs. time (days) of treatment in EMT6/CPR cell line. **(C)** Tumor sizes in EMT6/P cells after dissection on day 10 in all groups compared to each other. **(D)** Tumor sizes of EMT6/CPR cells after dissection at day 10 in all groups compared to each other. (1 = negative control; 2 = cisplatin; 3 = WSR; 4 = IF; 5 = WSR + IF; 6 = WSR + cisplatin; 7 = WSR + IF + cisplatin). Results are expressed as means ± SEM (*n* = 6). *P < 0.05, ^**^P = 0.001, ^***^P < 0.001. (*: treatment groups compared with the control group).

Generally, single treatment revealed a lower reduction in tumor size than combination treatments. The analysis showed that EMT6/P complied with the positive control (cisplatin) more than the WSR extract and IF as cisplatin reduced the tumor by 74.21% against 50% of mice with no detectable tumor. WSR extract and IF displayed approximately the same reduction in tumor size (51.97 and 52.75%, respectively) and the same curable rate (33.33%). In EMT6/CPR, the cells responded to WSR extract in a way better than the other single treatments by 40.49% lowering in tumor size and 50% curable rate. Fasting recorded a higher reduction in tumor size compared to cisplatin, with values of 37.22 and 18.80%, respectively, along with the same curable rate of 33.33%. Figures 6A,B illustrate the difference between treatments in tumor volume reduction at three time-points during treatments. The change in tumor size can be ascertained from Figures 6C,D, which show the final average volumes of the dissected tumors.

#### Effect of Treatments on Mice’s Average Weight

**FIGURE 7:**
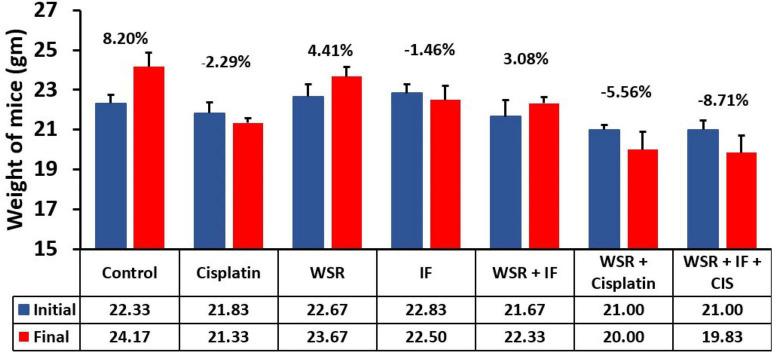
Mice average weight (gm) on days 1 and 10 in all groups compared with each other. (%): percentage of change in body weight. Results are expressed as means ± SEM (*n* = 6).

Concerning mice body weight, all treated groups registered weight loss except the WSR extract-treated group (25), and WSR/IF-treated group registered a significant weight gain compared with the IF-treated group, *p* < 0.001. Additionally, the control group recorded an increase in body weight (8.21%). Despite the effect of WSR extract on weight gain, cisplatin caused a non-significant weight loss compared to the control (8.20%) when administered either alone (–2.29%), along with WSR extract (–5.56%), or with WSR extract and IF (–8.71%; Figure 7).

#### Effect of the Treatments on Glucose and β-Hydroxybutyrate Levels

**FIGURE 8:**
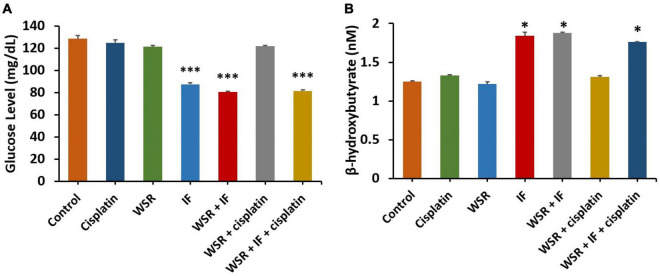
Effect of treatments on glucose and β-hydroxybutyrate (BHB). **(A)** Serum level of glucose for different treatments. **(B)** Serum level of BHB for different treatments. Results are expressed as means ± SEM (*n* = 3). *P < 0.05, ^***^P < 0.001. (*: treatment groups compared with the control group).

The subsequent analysis of glucose levels showed that treatments with IF had the lowest level of glucose either as a single treatment or in combination (*p* < 0.001). Generally, WSR extract-treated groups had lower glucose levels than the single treatment cisplatin or the control, and when we compared IF alone or along with WSR extract, nevertheless, the reduction was insignificant (Figure 8A). Additionally, we were interested to evaluate the comparative profiles between the treatment groups concerning their effect on the level of BHB. As expected, IF and its combination resulted in the highest values of BHB with a significant difference (*p* < 0.05) from the others (Figure 8B).

#### Safety Profile

**FIGURE 9:**
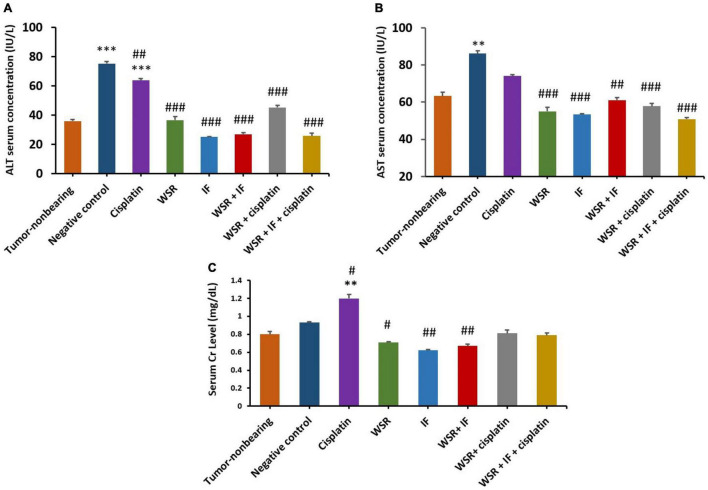
Safety profile for the treatments [WSR extract (100 mg/kg/d), cisplatin (5 mg/kg/week), IF, their combinations]. **(A)** Serum alanine aminotransferase (ALT) level measured by (IU/L). **(B)** Serum aspartate aminotransferase (AST) level measured by (IU/L). **(C)** Serum creatinine level measured by (mg/dl). Results are expressed as means μ SEM (*n* = 3). ^**^P = 0.001, ^***^P < 0.001. ^#^*P* < 0.05, ^##^*P* = 0.001, ^###^*P* < 0.001. (*: treatments group compared with the tumor-non-bearing group, ^#^: treatments groups compared to the negative control).

Alanine aminotransferase and AST assays were performed as they are considered markers for liver toxicity. Serum levels of the liver enzymes were measured for all treated groups with WS extract, IF, cisplatin, their combinations, the negative (untreated) control, and tumor nonbearing mice, which did not bear any tumors as a reference for liver function. The current study found that the levels of serum ALT are within the normal range for all treated groups compared with the normal-untreated mice, however, cisplatin-treated group recorded significantly higher ALT value. In turn, the cisplatin group recorded 63.88 IU/L of ALT, which is 1.77 times higher than the normal group, although combination groups that included cisplatin revealed better results with lower values of ALT levels (45.33 and 25.83 IU/L for cisplatin with WSR extract and for the triple combination with IF, respectively). Additionally, the effect of WSR extract, IF, and their combination on ALT levels were 36.66, 25.12, and 26.94 IU/L, respectively (Figure 9A). However, serum AST levels were normal for all treatment groups compared with the tumor nonbearing mice as they recorded an insignificant difference with *p* > 0.05. The cisplatin-treated group achieved a higher value than the tumor nonbearing mice (74.04 and 63.32 IU/L, respectively). However, this difference was insignificant (*p* = 0.195). Moreover, combination treatments, including cisplatin, registered lower values of AST (57.77 and 50.83 IU/L for cisplatin with WSR extract and the triple combination with IF, respectively; Figure 9B).

In the case of serum creatinine, normal levels of creatinine were observed between tumor nonbearing mice and the other mice groups, which were treated with the above-mentioned treatments. However, the single treatment of cisplatin increased creatinine levels significantly (1.2 mg/dl with a *p* value of 0.001). Note that combined treatment of cisplatin with either WSR extract or WSR extract and IF showed lower creatinine levels than cisplatin alone, with values of 0.81 and 0.79 mg/dl, respectively (Figure 9C).

## Discussion

Diverse obstacles hinder the successful treatment of breast cancer due to toxicity to normal cells, the narrow therapeutic index of chemotherapy, and MDR. The latter is a major obstacle because MDR has accounted for the failure of treatments and subsequently death (26). The combination of chemotherapy with natural products is widely used these days to overcome MDR (27). As another option, IF can reduce tumor incidence, potentiate the effectiveness of chemotherapy, and improve the response to chemotherapy (28). In this research, water extract of WSR and IF was examined compared with cisplatin *in vitro* using EMT6/P and EMT6/CPR breast cancer cell lines and *in vivo* using female Balb/C mice inoculated with EMT6/P and EMT6/CPR breast cancer cell lines. *In vitro* and *in vivo* tests showed promising results. It is worth mentioning that the WSR extract, IF combination, and triple combination have not been tested before.

*In vitro*, our results based on the viability assay indicated that WSR extract prevented EMT6/P and EMT6/CPR cell line viability in a concentration-dependent pattern. The present results agree with previous studies. Prasad et al. (29) observed a dose-dependent anti-breast cancer activity for the crude water extract of WSR on MCF-7 cell lines (29). This antiproliferative effect of the WSR extract is related to its content of anticancer components (Table 1). Based on the previous finding, succinic acid (68.52%) revealed an apoptotic effect on acute lymphoblastic leukemia (T-ALL cell line) and increased caspase-3 activity in human leukemic lymphoblasts (CCRF-CEM cell line) *in vitro* (30). Additionally, anthranilic acid (16.87%) has been widely used with its derivatives to fight cancer and it has an antiproliferative effect (31). Furthermore, the phenolic compounds, gallic acid (7.52%) can reduce viability and promote apoptosis (32) by upregulation of Fas and FasL and induction of p53 and caspase-3 (33). The latter is considered a key enzyme in the execution of apoptosis (5). Considering that WSR extract caused a dramatic increase in caspase-3 levels in both EMT6/P and EMT6/CPR cell lines, and that potentiates the apoptotic effect and reduce viability (Figures 5A,B). These outcomes were matched with a previous study where the caspase-3 activity was enhanced upon the use of WSR extract on MDA-MB231 (34).

Cisplatin was used as a positive control, along with the WSR extract. WSR extract with cisplatin showed a moderate synergism in both cell lines and reduced the used dose of cisplatin influentially, in addition to their ability to reduce the resistance fold of cisplatin (Table 2). Thus, it was concluded that WSR extract alongside cisplatin resulted in greater synergism than any other combination tested alone. Consistent with previous studies, withaferin A synergized the effect of paclitaxel on both drug-sensitive and drug-resistant NSCLC cells *in vitro* (35). Also, the antiapoptotic effect of this combination enhanced the level of caspase-3 effectively compared with the control, WSR extract-treated cells, and cisplatin-treated cells in both cell lines. As noted, WSR extracts remarkably potentiated the cisplatin response in the resistant cell line (Figures 5A,B), which means that WSR extract sensitized the resistant cell line to cisplatin at lower doses. Previously, Cohen et al. (36) showed that withaferin A along with sorafenib raised caspase-3 levels efficiently in papillary and anaplastic cancers (36).

Different treatments were tested *in vitro* in this research. In agreement with *in vitro* data, WSR extract reduced tumor size in EMT6/P and EMT6/CPR *in vivo* to 51.97 and 40.49%, respectively (Table 3). These findings follow previous studies where water extract of WS reduced tumor size in mice bearing cervical (HeLa) and colorectal (HT-29) cell-derived tumors (37). Another previous study observed that withaferin A, a component in WSR, showed tumor size reduction in mice injected with HeLa cells (38).

Another side of interest in this context is IF, which showed *in vivo* antitumor effect by reducing the tumor size in both EMT6/P and EMT6/CPR tumor-bearing mice by 52.75 and 37.22%, respectively (Table 3). It has been reported that IF reduced tumor size in a colon cancer xenograft mice model (CT26 cells) (39). Depending on that, the proposed antitumor effect of IF is due to reversing the Warburg effect and increasing BHB. Both have approved antitumor activity as mentioned before. Moreover, serum glucose level is an indicator for the Warburg effect (16), which was tested in this study. Based on the obtained results, cancer-induced mice that were treated with IF displayed a considerable serum glucose reduction compared with the control group (Figure 8A), which means that the Warburg effect was reversed and this was also proved before (40). The same groups had a higher level of BHB, which is considered an anticancer molecule (41) (Figure 8B).

Different combinations were explored *in vivo* in this research. First, combination treatment of WSR extract and cisplatin showed more size reduction of tumor (81.12 and 69.49% in mice bearing EMT6/P and EMT6/CPR cell lines, respectively; Table 3). These observations provided the basis that cisplatin and its combination with WSR extract resulted in greater efficacy and potency than the use of the drug alone in both cell lines. Interestingly, WSR extract sensitized the resistant cell line to cisplatin, remarkably.

Alternatively, a combination treatment of WSR extract and IF resulted in more tumor size reduction (60.52%) compared with a single treatment of WSR extract (51.97%) and IF (52.75%) in EMT6/P cells. Furthermore, combination treatment of WSR extract and IF resulted in more tumor size reduction (53.36%) compared with single treatment WSR extract (40.49%) and IF (37.22%) in EMT6/CPR cells. This combination has not been tested before; however, IF showed a synergistic effect with ascorbic acid vs. Kirsten rat sarcoma virus, a gene that makes a protein that is involved in cell signaling pathways that control cell growth, cell maturation, and cell death (KRAS) mutated cancers (42). Others observed that the combination of WS and Maitake (*Grifola frondosa*) extract had a synergistic effect on immunity (43). Moreover, WSR extract induced oxidative stress, which could potentiate the effect of IF by reversing the Warburg effect (44). WSR extract countered the effect of IF on mice’s weight significantly (Figure 7).

The mechanistic analysis provided in the literature demonstrates that WS can sensitize cancerous cells to chemotherapy through the apoptotic pathway, which is considered a predominant pathway for cisplatin-induced cell death. WS activates tumor suppressor p53, a fundamental element for inducting cisplatin-induced apoptosis and overcoming resistance (45). Primarily, A can block the autophagy flux in breast cancer cell lines MCF7 and MDA-MB-231, which is considered another method to reverse chemoresistance (46).

**FIGURE 10:**
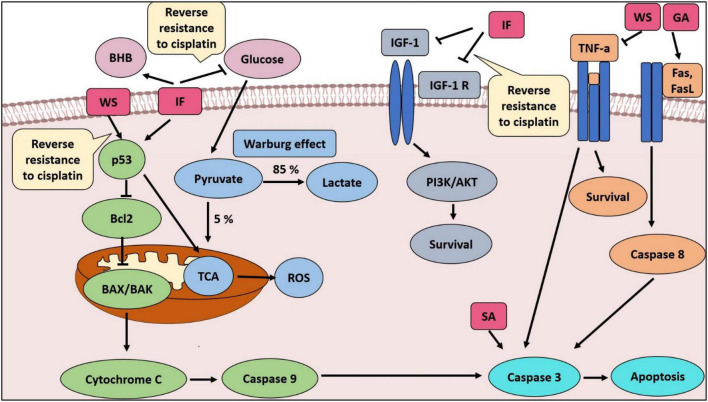
The proposed mechanisms for WSR extract and IF combination and how they can evade cancer and sensitize resistant cells to cisplatin, when added, based on the results obtained from this study and former studies. SA, succinic acid; GA, gallic acid; ROS, reactive oxygen species; TCA, tricarboxylic acid cycle.

It was imperative to examine the last combination, which included IF, WSR extract, and cisplatin. Further reduction in tumor size was detected by adding IF to the extract and cisplatin leading to complete vanishment of the tumor in the sensitive and resistant cell lines. That supported the synergistic effect of IF in both cell lines. Moreover, IF succeeded to sensitize the resistant cell lines to cisplatin and that was confirmed by the complete reduction of the tumor. Interestingly, this combination has not been evaluated in the literature (Figure 6; Table 3). Nevertheless, IF can reverse MDR by several pathways as revealed in the literature. The first way is by increasing p53, which is substantial for sensitization (47). By the same token, IF suppresses IGF-1 and IGF-1 receptors and that increases sensitivity to chemotherapy (48). Figure 10 illustrates the proposed mechanisms for WSR extract and IF combination and how they can evade cancer and can sensitize resistant cells to cisplatin, when added, based on the results obtained from this study and former studies.

The safety profile of anticancer agents is crucial to evaluating their toxicity. Liver enzymes (ALT and AST) and creatinine were used as indicators of liver and kidney functions, respectively. Results demonstrated that all treated groups had a normal level of ALT, except the cisplatin-treated group. As mentioned previously, cisplatin can induce hepatotoxicity. Combination treatments showed lower ALT levels than single treatment with cisplatin (Figure 9A). On top of that, AST levels were normal in the treated groups without exception. Nevertheless, combination treatments revealed lower AST levels than cisplatin alone (Figure 9B). In the literature, the extract of WSR showed hepatoprotective and antioxidant effects on radiation-induced hepatotoxicity (49). In contrast, creatine levels were normal in the treatment groups, excluding the cisplatin-treated group. The explanation for this finding is that cisplatin induces nephrotoxicity, which is one of the most serious obstacles that hinder cisplatin use (50). Despite that, combination treatment relieved the nephrotoxicity of cisplatin (Figure 9C). Formerly, water extract caused a remarkable elevation in the antioxidant activities of glutathione and superoxide dismutase to conserve renal tissue damage from gentamicin (51).

## Conclusion

Based on the data presented here, we concluded that the combination of WSR extract and cisplatin has a synergistic anticancer effect on both the parent and the resistant cell lines *in vitro* and *in vivo*, better than cisplatin alone through apoptosis induction and caspase-3 activation. On the other hand, the combination of IF with WSR extract has a superior ability to cause a reduction in tumor size. The activity of this combination was enhanced in the presence of cisplatin and caused complete tumor regression. These combinations are safer for the liver and kidney than the conventional therapy cisplatin. Such novel findings are worth the opportunity of expanding the range of research to establish better treatment for breast cancer in the future. Further studies are needed to evaluate the expression levels of antiapoptotic genes including Bcl2, BAX, and caspase 8 and to study morphological changes in cells after treatment.

## Data Availability Statement

The original contributions presented in the study are included in the article/supplementary material, further inquiries can be directed to the corresponding author.

## Ethics Statement

The animal study was reviewed and approved by Research and Ethical Committee of Applied Science Private University.

## Author Contributions

SJ: experimental work, data collection, data analysis, and wrote the original draft. WT: conceptualization, the direction of the work, supervision, data analyses, wrote revision, and editing. Both authors contributed to the article and approved the submitted version.

## Conflict of Interest

The authors declare that the research was conducted in the absence of any commercial or financial relationships that could be construed as a potential conflict of interest.

## Publisher’s Note

All claims expressed in this article are solely those of the authors and do not necessarily represent those of their affiliated organizations, or those of the publisher, the editors and the reviewers. Any product that may be evaluated in this article, or claim that may be made by its manufacturer, is not guaranteed or endorsed by the publisher.
